# Characterization of global wildfire burned area spatiotemporal patterns and underlying climatic causes

**DOI:** 10.1038/s41598-021-04726-2

**Published:** 2022-01-12

**Authors:** Ke Shi, Yoshiya Touge

**Affiliations:** Department of Civil and Environmental Engineering, Tohoku University, Sendai, Miyagi 980-8579 Japan

**Keywords:** Natural hazards, Hydrology

## Abstract

Wildfires are widespread disasters and are concurrently influenced by global climatic drivers. Due to the widespread and far-reaching influence of climatic drivers, separate regional wildfires may have similar climatic cause mechanisms. Determining a suite of global climatic drivers that explain most of the variations in different homogeneous wildfire regions will be of great significance for wildfire management, wildfire prediction, and global wildfire climatology. Therefore, this study first identified spatiotemporally homogeneous regions of burned area worldwide during 2001–2019 using a distinct empirical orthogonal function. Eight patterns with different spatiotemporal characteristics were identified. Then, the relationships between major burned area patterns and sixteen global climatic drivers were quantified based on wavelet analysis. The most significant global climatic drivers that strongly impacted each of the eight major wildfire patterns were identified. The most significant combinations of hotspots and climatic drivers were Atlantic multidecadal Oscillation-East Pacific/North Pacific Oscillation (EP/NP)-Pacific North American Pattern (PNA) with the pattern around Ukraine and Kazakhstan, El Niño/Southern Oscillation-Arctic Oscillation (AO)-East Atlantic/Western Russia Pattern (EA/WR) with the pattern in Australia, and PNA-AO-Polar/Eurasia Pattern-EA/WR with the pattern in Brazil. Overall, these results provide a reference for predicting wildfire and understanding wildfire homogeneity.

## Introduction

Wildfire, which is strongly responsive to climatic drivers, is a critical component of the natural earth system’s ecological process at scales ranging from local to global. Higher temperatures, more rain-free days, more wildfire events, and more wildfire-affected areas induce significant wildfire danger variations^1^. During 2001–2018, historical estimates of annual global wildfire burned areas ranged from 394 to 519 million hectares, with an average of 463 million hectares^2^. The Copernicus Sentinel-3 mission recorded 79,000 wildfires worldwide in August 2019 compared to just over 16,000 wildfires detected during the same period in 2018^3^. An apparent increase in catastrophic wildfires has been found globally in recent years. For example, California suffered wildfire in 2018 on the heels of a devastating 2017 wildfire^4^. In 2020, wildfires in the western USA were considered the most destructive wildfires in the USA. It is additionally estimated that there were thousands of smoke-related deaths, and over 10,000 structures have been damaged or destroyed^5^. In 2019, wildfires burned 1.01 million hectares in Alaska, and massive wildfires occurred in Siberia, which were both driving extremely high temperatures^4^. Between September 2019 and early January 2020, approximately 5.8 million hectares of mainly temperate broadleaf forest were burned in eastern Australia^6^, and these were the largest wildfires recorded in Australian temperate broadleaf forests^7^.

One of the critical factors affecting wildfires is climate. The Intergovernmental Panel on Climate Change states that “climate variability is often the dominant factor affecting large wildfires”^8^. The Global Climate Observing System^9^ defined wildfire disturbance as an “essential climate variable” and highlighted the need for long-term time series data to quantify the links between climate and wildfire. In particular, the globalizing effect of the atmosphere occurs by synchronizing wildfire weather conditions at distant locations via teleconnection mechanisms induced by climatic drivers^10^, emphasizing the importance of studying synchronous wildfires. One of the best known of these mechanisms is the El Niño-Southern Oscillation (ENSO)-wildfire dynamic in Insular Southeast Asia^11^. Strong correlations were found between wildfires and the ENSO index (Southern Oscillation Index and Niño 3.4 index) in equatorial forests (5.5°S–5.5°N)^11^. The impact of the Arctic Oscillation (AO) pattern on interannual wildfire variability in Central Siberia was found by Balzter^12^. A linear combination of the Arctic Oscillation index and the summer temperature could largely simulate the burned area in Central Siberia^12^. Years of combined positive Atlantic Multidecadal Oscillation (AMO) and negative ENSO and Pacific Decadal Oscillation (PDO) phases represent “triple whammies” that significantly increased the occurrence of drought-induced wildfires in western Colorado, USA^13^, which provided evidence that the recent shift to the positive phase of the AMO would promote higher wildfire frequencies in Colorado^13^. However, it turns out that compared with higher wildfire frequencies, the increasing trend of higher severity and larger burned area wildfires in the southwestern USA is more significant^14^, emphasizing our concern about wildfire burned areas.

Overall, climate variability could influence wildfire behavior and account for the variability in wildfire severity at various temporal and spatial scales ^15^. Therefore, substantial research has focused on the relationship between climate weather and wildfire at local levels. Aldersley et al. analyzed the relationship between the burned area and meteorological elements such as high temperature and intermediate annual rainfall in fourteen global subcontinental regions^16^. Hantson et al. used a generalized additive model (GAM) to explore the links between the global burned area and vegetation productivity, precipitation, and essential variables in a 2° grid^17^. However, these studies mainly focused on gridpoint-specific meteorological elements and wildfire burned area relationship analysis. Global climatic drivers such as AMO and ENSO were not considered from a more in-depth perspective in their global wildfire study. Indeed, global climatic drivers are often the most fundamental factor that affects meteorological elements such as precipitation and temperature, which in turn affects global wildfires. However, there is still a gap in the comprehensive understanding of the relationship between global climatic drivers and wildfires, and even finding the teleconnections between different regional wildfires can be expected, implying that the same global climatic driver affects wildfires in several regions.

Indeed, the teleconnections between global climatic drivers and multiple hydrometeorological variables in different regions have attracted the attention of researchers in recent years. Fifteen regional hazards (such as Australian windstorms, rainfall in China and rainfall in the USA) shared connections via ENSO^18^. The Indian Ocean Dipole, North Atlantic Oscillation, and Southern Annular Mode were secondary sources of significant teleconnection^18^. This kind of research could strengthen the understanding of the global likelihood of concurrent hazard occurrence. Su et al.^19^ analyzed the multivariate relationship between the streamflow of sixteen large rivers in the world and meteorological factors/global climatic drivers based on wavelet coherence, which provided a reference for medium- to long-range hydrological forecasts. In addition, Nguyen et al.^20^ examined the combined effects of ENSO and PDO on global droughts and found that when ENSO and PDO are in phase, drought tends to intensify and expand in the Amazon, India, Central China, Indonesia and eastern Australia. This teleconnection analysis between global climatic drivers and hydrometeorological variables could be used to explore the potential connections in regional climates. However, there is still a lack of understanding of the climate connections of global wildfire patterns. To better understand the spatiotemporal changes and teleconnection of wildfires in different regions, Page et al.^10^ provided the first global assessment of spatiotemporal wildfire variability and cross-regional climate influence. The limitation of these studies was that they focused only on the number of wildfire events and selected only ENSO as a global climatic driver for analysis. Another key factor for measuring wildfire is the burned area, reflecting the extent of wildfire severity^21^. Burned area is widely used to measure global wildfire characteristics, which has been linked to paleorecords^22^ and is used to calculate fluxes of carbon from the biosphere to the atmosphere^23^. However, wildfires are often affected by multiple global climatic drivers at the same time^16^, and the adoption of more global climatic drivers will further strengthen the understanding of the influence of climate on wildfires.

Until now, previous studies have not filled gaps in exploring the spatiotemporally homogeneous regions of global burned areas and multiple climatic influences. Therefore, a comprehensive study of global wildfire patterns will help us better understand global wildfires. Considering that the causes of wildfires are intricate and usually influenced by multiple factors, it is essential to study multivariable coherence to better reveal the salient features of wildfires from a global perspective. Overall, the main aim of this study was to analyze the relationships between major wildfire patterns and various global climatic drivers. First, the distinct empirical orthogonal function (DEOF) was applied to identify the spatiotemporally homogeneous regions of burned area around the world. The cross wavelet transform (XWT) and wavelet coherence (WCO) were used to analyze the relationships between wildfire burned area in major patterns and various global climatic drivers based on global burned area patterns. Then, by determining the common regions and common global climatic drivers in different patterns, the relationship between specific regions and significant global climatic drivers was discussed to verify the results of the teleconnection analysis.

## Results

### Spatial and temporal patterns of wildfire based on DEOF

The DEOF calculation used the log*BAA* (log-transformed burned area anomalies) time series of each 1° × 1° grid cell on a monthly scale. The first eight DEOFs represented 30% of the total variance. There are two main reasons why the explained variance does not reach high values. On the one hand, our analysis of temporal and spatial patterns of burned area is based on a global scale. The competition between intraregional correlation and teleconnections between two distant regions would make it difficult to find the dominant patterns with high explained variance, such as regional analysis. For example, in the spatial and temporal analysis of global drought patterns from Dai et al.^24^, the explained variance of PC1 and PC2 is also only 6.7% and 5.1%, respectively. On the other hand, wildfires are affected by multiple factors, not only climate but also human activities. The differences between regions are more significant than traditional hydrometeorological variables. Similarly, in the global spatial and temporal analysis of the number of fires from Page et al.^10^, the first nine patterns represented 40% of the total variance. Considering that the causes of the burned area are more complicated than the number of fires, the first eight DEOFs accounting for 30% are actually in conformity with the expected results. It should be noted that since this study focuses on wildfire anomalies, the wildfire anomalies in wildfire-prone regions were less impacting. For example, the USA, a country with frequent wildfires almost every year, did not receive any attention in the wildfire homogeneous region analysis of this study.

**Figure 1:**
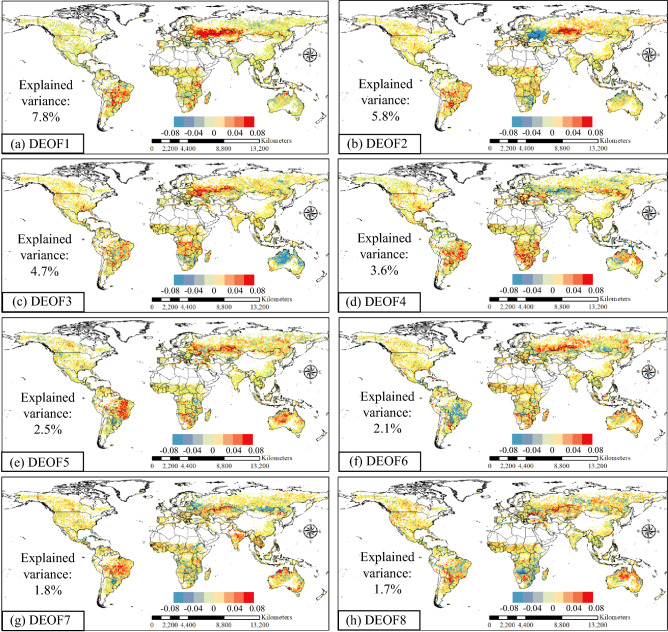
DEOF1–8 for the spatial distribution of log*BAA.* This figure was generated using ArcGIS version 10.1 (https://www.esri.com/en-us/home). The data used in the calculation comes from Fire CCI v5.1 dataset (https://geogra.uah.es/fire_cci/firecci51.php). Shape for countries of the world downloaded from Geografía, SIG y Cartografía Digital. (http://tapiquen-sig.jimdofree.com).

**Figure 2:**
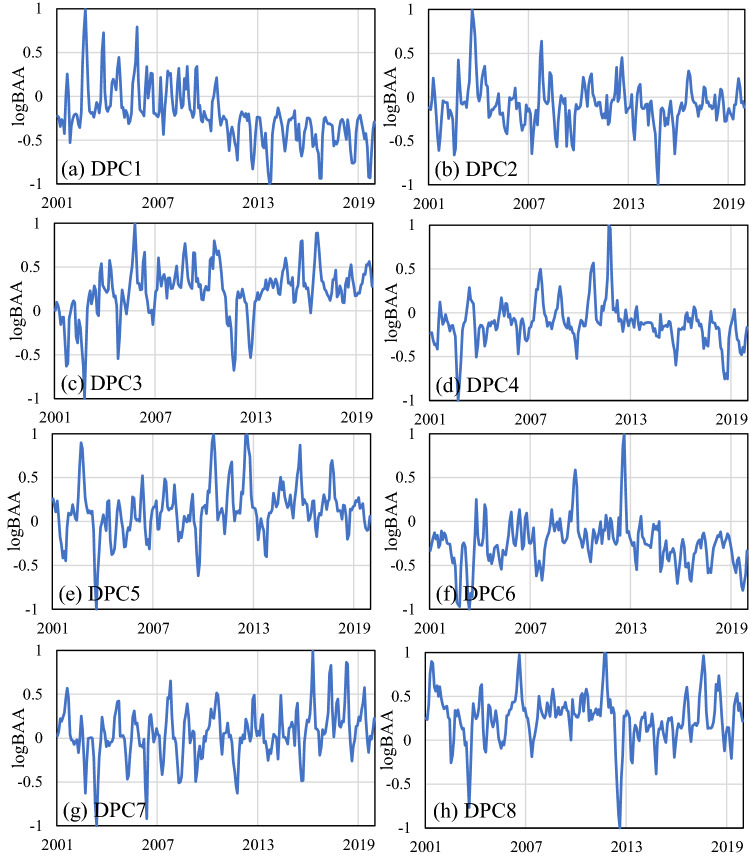
DPC1–8 time series of log*BAA* (DPCs are the projection of log*BAA* time series in each DEOF pattern)*.* The data used in the calculation comes from Fire CCI v5.1 dataset (https://geogra.uah.es/fire_cci/firecci51.php).

Figure 1 displays the spatial patterns of DEOF1–8, while the temporal part of DPCs is shown in Fig. 2. The variation range of the DPCs is different. To show the time series more clearly, the DPCs are normalized to the range of − 1 to 1. DPCs are the projection of log-transformed BAA time series in each DEOF pattern. The positive value of DPCs indicated that the burned area in the positive loading region was more than the multiyear monthly average, while the burned area in the negative loading region was less than the multiyear monthly average.

Different DEOFs represented different abnormal characteristics of wildfire burned areas. Specifically, the top 20% of the largest (smallest) DEOF values are considered high positive (low negative) loading values. For example, in DEOF2, the spatial distribution illustrated that low negative loadings occurred in Part of Russia and Ukraine. Meanwhile, the high positive loadings were mainly concentrated in northern Kazakhstan, which also meant that these regions had opposite characteristics as those of the negative loading regions.

For the time function, the particular time when the wildfire occurred abnormally could be found from DPCs. There was a significant decreasing trend in DPC1, which meant that the burned area in part of Russia and north of Kazakhstan, where high positive loadings were mainly concentrated, was decreasing. This decreasing trend was also found in the study of spatiotemporal variation in the burned area in Kazakhstan from Xu et al.^25^. In contrast, for DPC7, the burned area in recent years was larger than before, indicating that the burned area in eastern Brazil was increasing. This increasing trend was also found in the research of Forkel et al.^26^. For specific wildfire events, in DPC1, there was a significant abnormally high value in August 2002. Correspondingly, in 2002, Kazakhstan recorded the most severe wildland fire, with a maximum burned area of 4.6 million hectares since 1997^25^. For DPC5, an unusually high value appeared in 2010 and 2012, and high positive loading was found in Brazil. These two huge wildfires only two years apart in Brazil were also discovered by Schmidt et al.^27^

**Figure 3:**
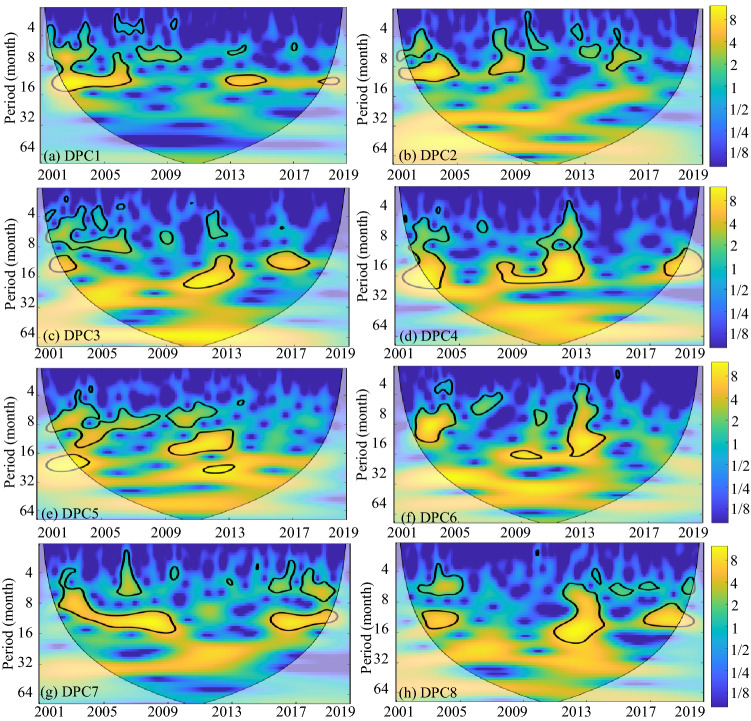
WPS of the DPC time series (Periodicity of time series). The black contour designates the 95% confidence level against red noise, and the COI, where edge effects might distort the picture, is shown as a lighter, paler shade. This figure was generated using MATLAB version R2020b (https://jp.mathworks.com/). The data used in the calculation comes from Fire CCI v5.1 dataset (https://geogra.uah.es/fire_cci/firecci51.php).

The specific temporal periodicity of DPC1–8 identified by CWT is displayed in Fig. 3. The black contour designates the 95% confidence level against red noise, and the COI, where edge effects might distort the picture, is shown as a lighter, paler shade. These CWT results suggested that none of the DPCs seemed to have a long-term dominant periodicity. Except for DPC1 and DPC4, all DPCs showed dominant but intermittent approximately annual (8–16 months) periodicities compared to interannual periodicities. In particular, there were two consecutive annual periodic bands of 2003–2009 and 2016–2018 in DPC7. The periodic bands of DPC2 tended to be concentrated at the interannual scale, while the periodic bands of DPC4 tended to be concentrated at the multiyear scale.

### Teleconnection between global climatic drivers and DEOFs

The effects of global climatic drivers on DPC1–8 are summarized in Table 1. To highlight the strong drivers among all the global climatic drivers of each DEOF pattern, the global climatic drivers with the largest PASC are italics, and the top three PASCs are shown in bold. It was evident from the PASC results that different global climatic drivers made different contributions to the wildfire burned area patterns. The highest PASC for climatic drivers ranged from 11.3 to 21.0% across DPC1–8, and the average highest PASC was 16.0%. Among these global climatic drivers, PNA and EA/WR had the broadest ranges of influence on DPC1–8 since these three factors all affected the three distributions of DPCs. Simultaneously, the AAO and DMI were the global climatic drivers that had no decisive effect detected in any DEOF1–8 patterns.

**Table 1:** PASC (%) for the wavelet transform coherence between DPCs and global climatic drivers.

	DPC1 (%)	DPC2 (%)	DPC3 (%)	DPC4 (%)	DPC5 (%)	DPC6 (%)	DPC7 (%)	DPC8 (%)
TSA	9.5	3.5	10.4	14.5	1.3	**6.4**	3.3	7.0
POL	11.5	3.7	3.6	5.5	3.8	***15.0***	**11.4**	1.5
WP	6.5	7.9	12.4	8.0	4.7	4.5	8.1	12.0
PDO	4.8	8.4	12.3	**20.6**	6.5	5.2	8.4	4.1
ONI	4.6	8.4	12.2	17.3	4.6	**6.9**	5.8	6.6
AMO	***20.4***	4.9	5.9	10.0	6.8	4.7	**10.1**	6.8
DMI	3.6	3.8	9.4	5.9	2.6	4.1	5.0	4.7
AO	3.3	4.0	7.3	4.8	***11.3***	5.5	5.2	**13.7**
NAO	4.8	3.6	11.2	9.7	**10.6**	5.7	8.9	3.6
PNA	7.8	**12.5**	**15.0**	***21.0***	3.5	5.2	4.7	7.6
AAO	13.5	7.4	10.1	6.2	5.7	3.3	5.2	7.0
EA	5.6	**14.5**	4.4	11.9	4.3	3.8	3.9	7.1
EA/WR	4.9	10.0	8.3	6.8	**8.8**	4.0	***12.7***	***14.7***
TNA	**13.8**	3.5	**12.8**	10.6	4.7	5.7	6.2	9.3
MEI	5.4	5.9	***17.0***	**18.1**	2.8	5.4	4.5	4.7
EP/NP	**17.9**	***16.0***	8.3	14.8	7.0	6.1	4.1	**9.8**

Italic indicates the most significant global climatic driver, and bold indicates the first three most significant global climatic drivers.

**Figure 4:**
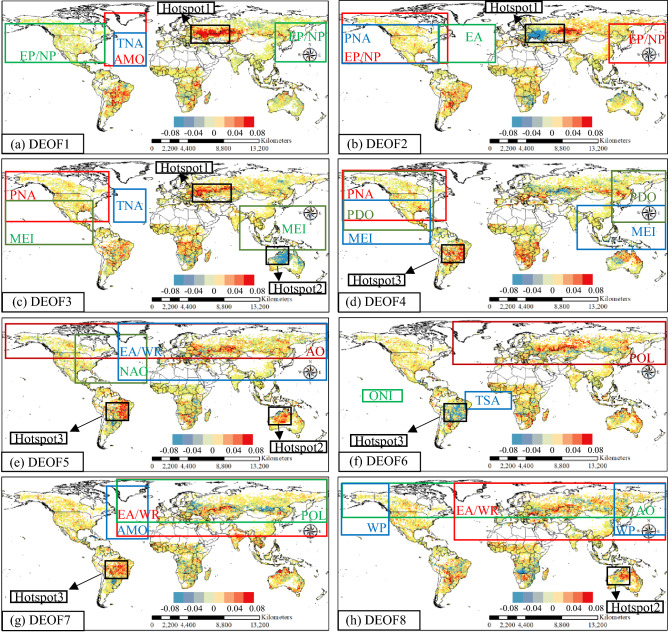
The location distribution of the top three global climatic drivers with the strongest influence on DEOF patterns. The red, blue and green rectangles indicate the strongest, second-strongest and third-strongest global climatic drivers on the DEOFs, respectively. The black circle indicates the common region in different patterns. Hotspot 1: around Ukraine and Kazakhstan; Hotspot 2: Australia; Hotspot 3: Brazil. This figure was generated using ArcGIS version 10.1 (https://www.esri.com/en-us/home). The data used in the calculation comes from Fire CCI v5.1 dataset (https://geogra.uah.es/fire_cci/firecci51.php) and NOAA (https://psl.noaa.gov/data/climateindices/list/). Shape for countries of the world downloaded from Geografía, SIG y Cartografía Digital. (http://tapiquen-sig.jimdofree.com).

Figure 4 shows the defined region of the three global climatic drivers that have the most significant influence on DEOF1–8. Some regions frequently appeared in different DEOF patterns. Hotspot-1 (around Ukraine and Kazakhstan) was found in DEOF1–3. Although the three most dominant global climatic drivers changed with the DEOF patterns, AMP, EP/NP and PNA were the strongest influencing drivers among these five climatic drivers, indicating that these three global climatic drivers had a relatively strong impact on hotspot-1. For DEOF3, DEOF5 and DEOF8, there were three different combinations of global climatic drivers affecting hotspot-2 (Australia): MEI-DEOF3, AO-DEOF5 and EA/WR-DEOF8. The global climatic drivers that affect hotspot 3 (Brazil) have become very diverse, where they were found to be affected by ten different global climatic drivers. Affected by ten climatic drivers, PNA, AO, POL and EA/WR were the dominant global climatic drivers in hotspot 3 of DEOF4–7.

**Figure 5:**
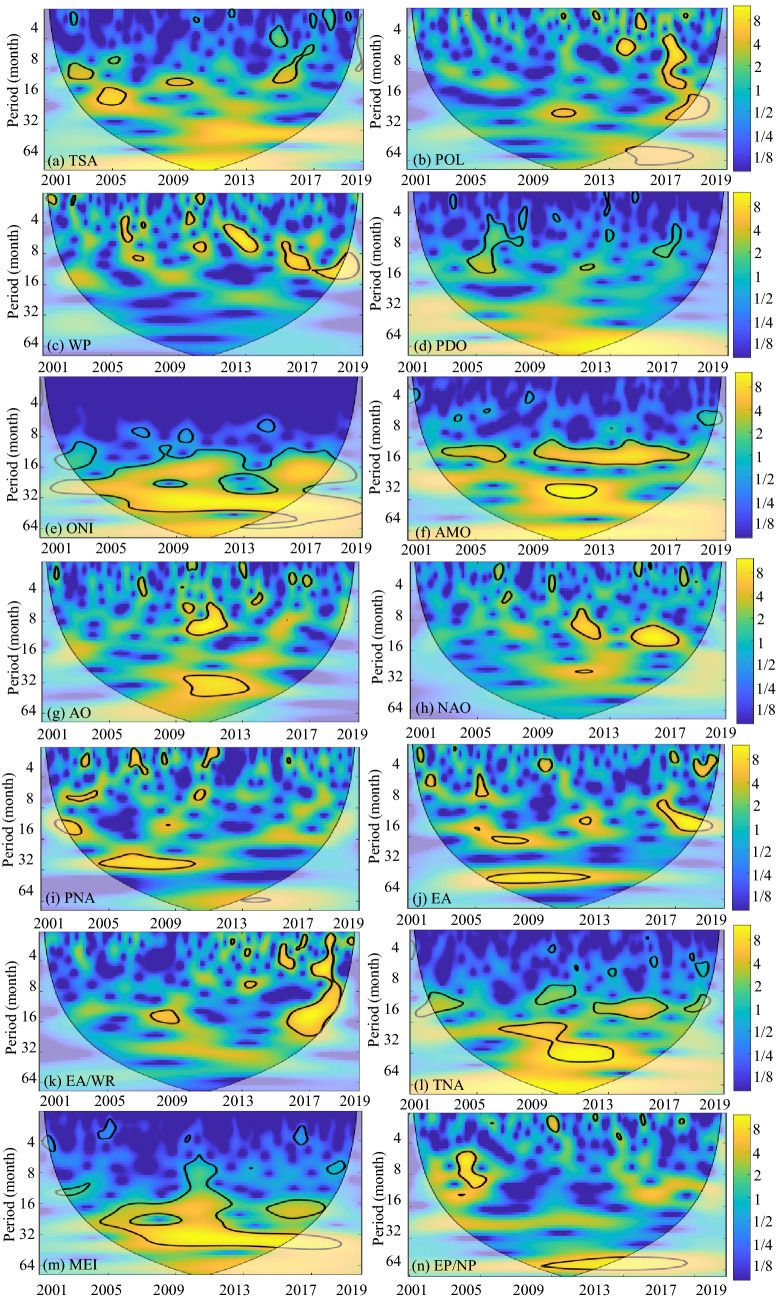
WPS of the large-scale climatic single time series (periodicity of time series). The black contour designates the 95% confidence level against red noise, and the COI, where edge effects might distort the picture, is shown as a lighter, paler shade. (DMI and AAO, which have no significant impact on any DEOFs, are removed). This figure was generated using MATLAB version R2020b (https://jp.mathworks.com/). The data used in the calculation comes from Fire CCI v5.1 dataset (https://geogra.uah.es/fire_cci/firecci51.php) and NOAA (https://psl.noaa.gov/data/climateindices/list/).

**Figure 6:**
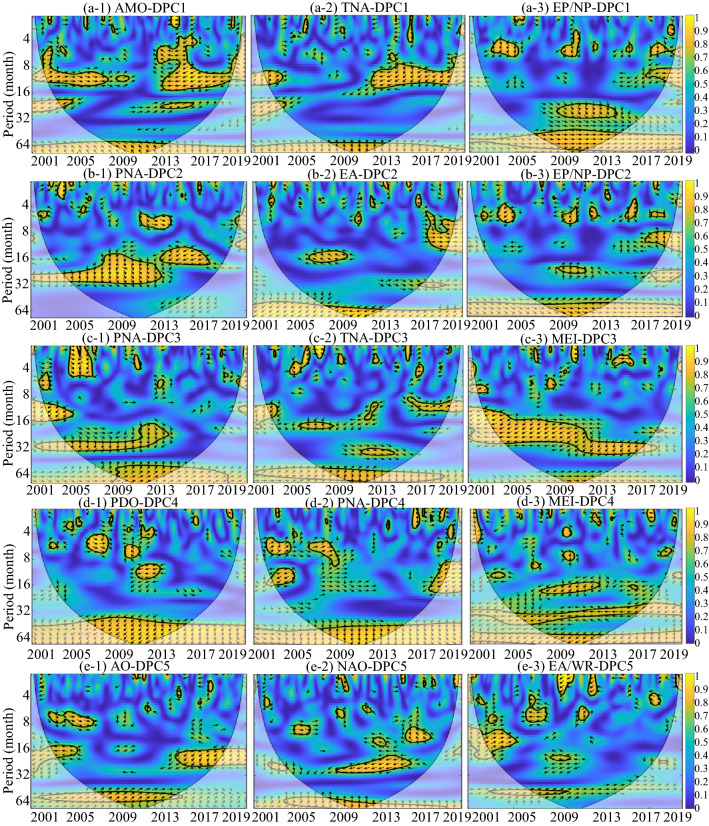
Squared wavelet coherence between the global climatic drivers and the temporal patterns of DPC1–5 (coherence coefficient between the global climatic drivers and DPCs). The black contour designates the 95% confidence level against red noise, and the COI, where edge effects might distort the picture, is shown as a lighter, paler shade. In addition, the phase lags are illustrated by black arrows. The y-axis represents the coherence period, and the color represents the level of the coherence coefficient. This figure was generated using MATLAB version R2020b (https://jp.mathworks.com/). The data used in the calculation comes from Fire CCI v5.1 dataset (https://geogra.uah.es/fire_cci/firecci51.php) and NOAA (https://psl.noaa.gov/data/climateindices/list/).

**Figure 7:**
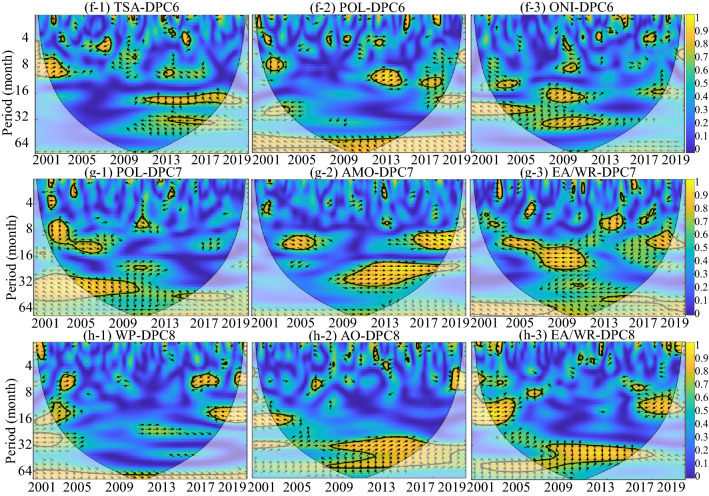
Squared wavelet coherence between the global climatic drivers and the temporal patterns of DPC6–8 (coherence coefficient between the global climatic drivers and DPCs). The black contour designates the 95% confidence level against red noise, and the COI, where edge effects might distort the picture, is shown as a lighter, paler shade. In addition, the phase lags are illustrated by black arrows. The y-axis represents the coherence period, and the color represents the level of the coherence coefficient. This figure was generated using MATLAB version R2020b (https://jp.mathworks.com/). The data used in the calculation comes from Fire CCI v5.1 dataset (https://geogra.uah.es/fire_cci/firecci51.php) and NOAA (https://psl.noaa.gov/data/climateindices/list/).

Figure 5 shows the temporal periodicity of sixteen global climatic drivers. The global climatic drivers ONI and MEI, which express the impact of ENSO in different ways, show significant multiyear periodicity. The dominant periodic band of the AMO is the intra-annual periodicity. However, it is difficult to find the dominant periodicity from the remaining thirteen global climatic drivers. Figures 6 and 7 show the coherence coefficient between DPCs and the top three global climatic drivers with phase lags between components, as illustrated by black arrows. Among DPC1–8, DPC6 showed the weakest coherence with global climatic drivers, with the top three average PASCs of 9.5%. DPC4 had 19.9% of the top three average PASCs, indicating that it had the strongest coherence with these climatic drivers.

For DPC1 and DPC4, there was high wavelet coherence at more than four-year scales (48–64 months) throughout the entire research period, indicating the dominant effect of the climatic drivers on the BAA of DPC1 and DPC4 on the multiyear timescale. Additionally, similar high wavelet coherence across the research period was also observed in EA-DPC2, EP/NP-DPC2, POL-DPC6 and WP-DPC8. At the same time, PNA-DPC2, PNA-DPC3, MEI-DPC3, TSA-DPC6 and AMO-DPC7 were typical of high wavelet coherence at approximately two-year scales (16–32 months) with global climatic drivers. This two-year wavelet coherence lasted the longest between MEI and DPC3, from 2001 to 2018, while the coherence between TSA and DPC6 only appeared during the period from 2010 to 2019.

**Figure 8:**
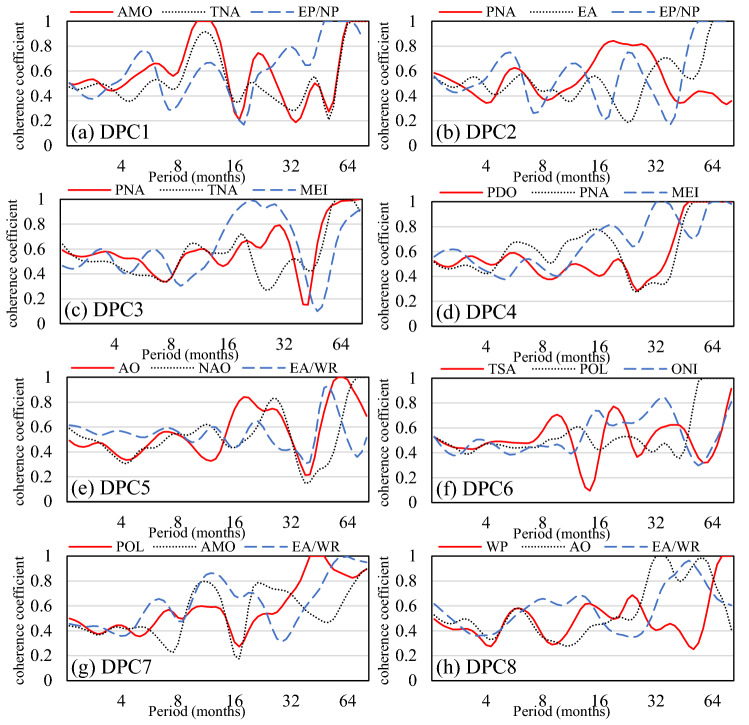
The global coherence coefficient between global climatic drivers and the temporal patterns of DPC1–8. The data used in the calculation comes from Fire CCI v5.1 dataset (https://geogra.uah.es/fire_cci/firecci51.php) and NOAA (https://psl.noaa.gov/data/climateindices/list/).

Figure 8 shows the global coherence coefficients, providing an evaluation of averaged coherence between monthly DPCs and the top three global climatic drivers over different timescales. By plotting the top three indices together, it becomes possible to compare the relative coherence significance of each index in each logBAA pattern under all-time scales. Only DPC1 had high coherence coefficients with global climatic drivers on the annual scale and multiyear scale simultaneously. For DPC3, there were high global high coherence coefficients on an approximately 2-year scale and more than a 4-year scale. These results indicated that certain global climatic drivers could have strong effects on both large and small time scales. However, other DPCs only showed high coherence coefficients on time scales larger than 32 months. In particular, some global climatic drivers did not reach high global coherence coefficients on all time scales, such as PNA-DPC2 and ONI-DPC6, indicating that these global climatic drivers have only limited impacts on these DEOF patterns.

**Figure 9:**
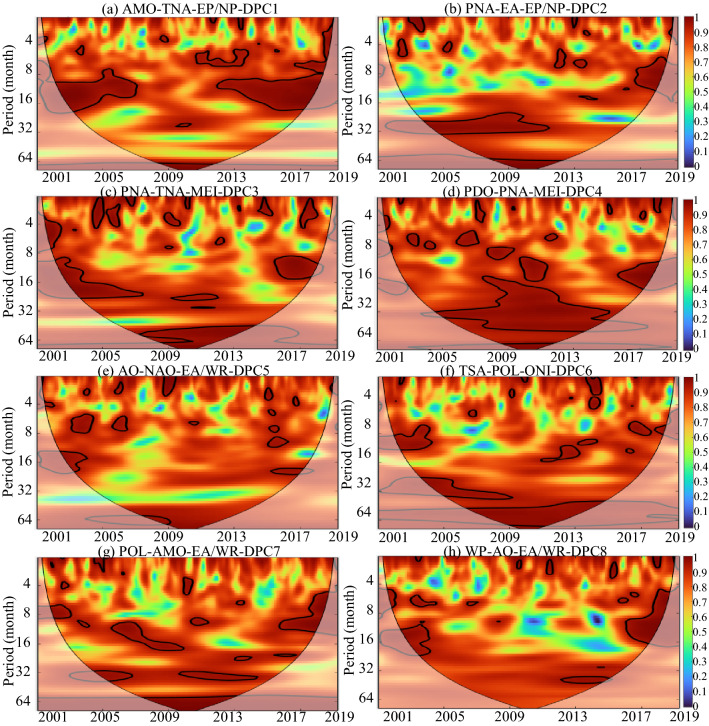
Three-factor multiple wavelet coherence between the top three global climatic drivers and the temporal patterns of DPC1–8. The black contour designates the 95% confidence level against red noise, and the COI, where edge effects might distort the picture, is shown as a lighter, paler shade. In addition, the phase lags are illustrated by black arrows. The y-axis represents the coherence period, and the color represents the level of the coherence coefficient. This figure was generated using MATLAB version R2020b (https://jp.mathworks.com/). The data used in the calculation comes from Fire CCI v5.1 dataset (https://geogra.uah.es/fire_cci/firecci51.php) and NOAA (https://psl.noaa.gov/data/climateindices/list/).

Figure 9 summarizes the combinations of the top three climatic drivers that explain the variations in global burned area patterns. From Fig. 9a, the interactive effect of AMO-TNA on DPC1 at 8–16 months and the interactive effect of AMO-TNA-EP/NP on DPC1 at 64 months can be identified. Nevertheless, for DPC9 (Fig. 9h), only WP showed significant coherence at 64 months, and the interaction effect of WP-AO-EA/WR on DPC1 became insignificant at 64 months. As a consequence, when climatic drivers show the same periodicity or have significant impacts on DPCs at the same time frequency, the interactive effect of climatic drivers will strengthen this influence relationship on DPCs. Conversely, when the interaction between climatic factors is not significant, multifactor coherence will reduce their impact on DPCs.

## Discussions

Overall, our teleconnection analysis has revealed how global climatic drivers affect global burned area patterns, which provides a possibility for informing the anticipation of burned areas. For global burned area patterns, regionality is greatly evident. These hotspots, such as around Ukraine and Kazakhstan, Australia and Brazil, frequently appear in different global burned area patterns. Similarly, around Ukraine and Kazakhstan and part of Australia were identified as hotspots of joint occurrence of wildfires and heatwaves^28^. Additionally, in the maximum monthly burned area fraction during 2001–2018 mapped by Sungmin et al.^29^, most of the regions were consistent with the identified hotspots in this study. Even though the Ukrainian region did not show a large, burned area, it showed significant burned area anomalies. Similarly, the United States, where wildfires frequently occurred, was also insignificant in our global burned area patterns.

For relationships between wildfire and global climatic drivers, the most significant combinations of hotspots and climatic drivers were AMO-EP/NP-PNA with the pattern around Ukraine and Kazakhstan, ENSO-AO-EA/WR with the pattern in Australia, and PNA-AO-POL-EA/WR with the pattern in Brazil. Actually, there have been studies proving how global climatic drivers affect specific regions.

**Figure 10:**
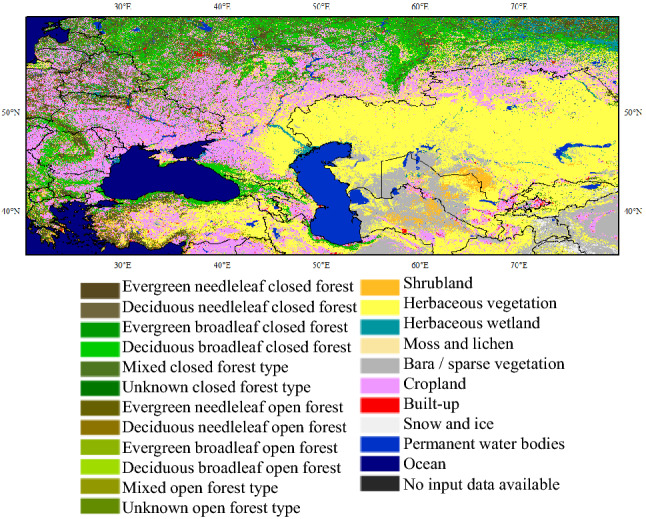
Land cover map of hotspot-1 (around Ukraine and Kazakhstan). This figure was generated using ArcGIS version 10.1 (https://www.esri.com/en-us/home). Shape for countries of the world downloaded from Geografía, SIG y Cartografía Digital. (http://tapiquen-sig.jimdofree.com). The land cover data was from Buchhorn et al.^32^ (https://land.copernicus.eu/global/products/lc).

Figure 10 shows the land cover of hotspot 1 (Ukraine and Kazakhstan). It can be seen that the Ukrainian region is covered by a large amount of cropland, which is scattered with different types of forests. Furthermore, the abandoned cropland in Ukraine has continued to increase since the dissolution of the Soviet Union^30^. These kinds of abandoned croplands are especially prone to wildfires because the vegetation rapidly develops, accumulating fuel^31^. However, it should be noted that the 1° resolution used in the global wildfire pattern analysis will cause cropland, abandoned cropland, and forest to appear in the same grid easily. For this uncertainty, log-transformation and processing of burned area anomalies will reduce the impact of humans on cropland burning as much as possible and focus on uncontrolled wildfires in abandoned cropland and forest areas.

For the combination of AMO with the pattern around Ukraine and Kazakhstan, on the annual timescales, a significantly higher sea level pressure and geopotential height in the summer of Eurasia will be observed after a previous warm winter AMO^34^. The high sea level pressure associated with descending atmospheric motion could lead to adiabatic warming^35^, increasing the probability of favoring fewer clouds and precipitation, further leading to more incoming solar energy and enhancing the warming rate^34^. Extreme high temperatures will further enhance the risk of extreme wildfires. However, there is a limitation of this study; that is, due to the availability of global wildfires, the results of large time scale coherence between AMO and wildfires may be suspicious on longer time scales. In fact, AMO itself has a long-famous periodicity characteristic of more than 60 years^36,37^. The AMO had a significant coherence with European temperature on a decades scale^32^. As the time series of wildfire data extends, larger-scale coherence between the AMO and wildfire is more likely to be detected.

**Figure 11:**
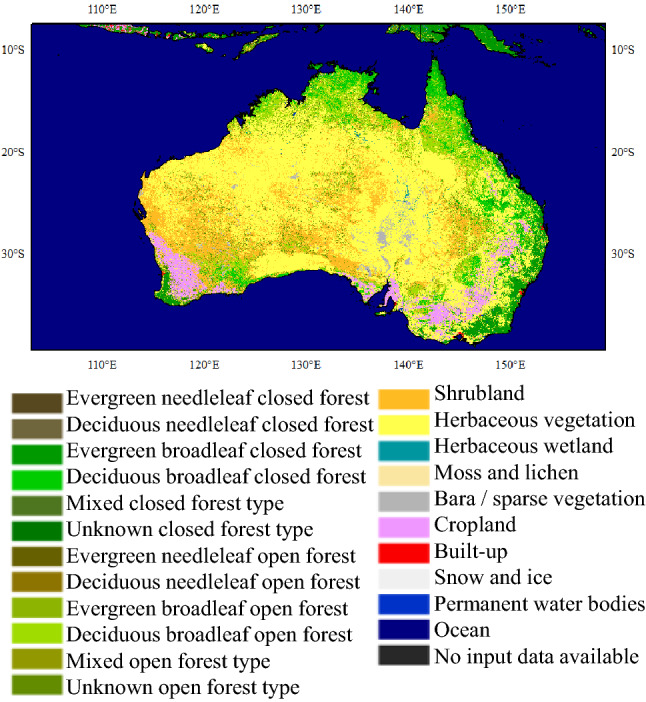
Land cover map of hotspot-2 (Australia). This figure was generated using ArcGIS version 10.1 (https://www.esri.com/en-us/home). Shape for countries of the world downloaded from Geografía, SIG y Cartografía Digital. (http://tapiquen-sig.jimdofree.com). The land cover data was from Buchhorn et al.^32^ (https://land.copernicus.eu/global/products/lc).

For the combination of ENSO with Australia, a strong relationship between ENSO events and burned area has already been found in northern Australia^38^. However, unlike forest wildfires in southeastern Australia^39^, grassland wildfires are more common in northern Australia^40^, as shown in Fig. 11. Large amounts of grassland in northern Australia provide fuel for wildfires. There is a famous grass-wildfire cycle in northern Australia^41–43^. The wildfire destroyed the vegetation, but the precipitation allowed new vegetation to grow in the charred areas. It is easy to recognize the importance of precipitation for the grass-wildfire cycle in northern Australia. Considering the apparent relationship between ENSO events and precipitation in northern Australia^38^, ENSO events tended to affect wildfire anomalies by controlling precipitation in northern Australia.

**Figure 12:**
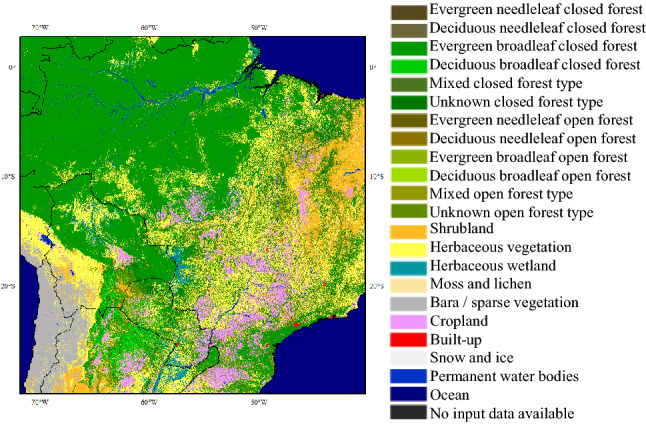
Land cover map of hotspot-3 (Brazil). This figure was generated using ArcGIS version 10.1 (https://www.esri.com/en-us/home). Shape for countries of the world downloaded from Geografía, SIG y Cartografía Digital. (http://tapiquen-sig.jimdofree.com). The land cover data was from Buchhorn et al.^32^ (https://land.copernicus.eu/global/products/lc).

As shown in Fig. 12, the vegetation types in hotspot-3 are dominated by broadleaf forests, shrubland, and herbaceous vegetation, while cropland only accounts for a small percentage. Compared with the widespread tropical rain forests in northern Brazil, Mato Grosso, Mato Grosso do Su, Tocantins, and Goiás with mixed vegetation types in central Brazil showed more burned area anomalies. Especially in Mato Grosso, the largest wildfire outbreaks occur in regions with illegal deforestation^44^. Despite this, RA Silvestrini et al. found that the wildfire risk in Brazil was more directly impacted by climatic than anthropogenic factors because favorable weather conditions are a precondition for human-induced wildfires^45^.

As for combination of PNA with Brazil. In particular, from March to May, equatorial central and eastern Pacific sea surface temperature anomalies were negatively correlated with precipitation over Northeast Brazil^46^. There are two main mechanisms by which Pacific sea surface temperature anomalies affect precipitation over Northeast Brazil: one is a direct mechanism through anomalous Walker circulation that influences Atlantic Intertropical Convergence Zone positioning^46,47^; the other is through an indirect mechanism via an atmospheric bridge similar to the PNA pattern, which affects tropical North Atlantic sea surface temperature anomalies, altering Atlantic Intertropical Convergence Zone positioning^48^. The Atlantic Intertropical Convergence Zone is often accompanied by cumulus clouds and heavy precipitation^49^. In other words, PNA can affect the wildfire risk of hotspot-3 by affecting precipitation anomalies.

Overall, this study establishes the teleconnection between global climatic drivers and burned area patterns for the first time and explores the physical mechanism behind their teleconnection from a global perspective. Under the combined influence of AMO and increased abandoned cropland, the hotspot-1 (around Ukraine and Kazakhstan) has shown the characteristics of significant burned area anomalies. The burned area of hotspot-2 (Australia) with grass-fire cycle changes with the inter-annual variation of ENSO. In hotspot-3 (Brazil), weather conditions controlled by the PNA are still a precondition for human-induced wildfires. The results of this paper are conducive to a better understanding of the spatiotemporal characteristics of global wildfire burned areas. Due to the changing climate, atmosphere, and ocean scenarios, focusing on global climate drivers provides an efficient and promising reference for predicting wildfire burned area anomalies. The conclusions will also be valuable for wildfire management, wildfire prevention, and global wildfire ecological climatology.

The study's limitations include the lack of validation of the physical mechanism between wildfires and global climatic drivers. Furthermore, some regions were ignored in our identified global wildfire patterns. Although some studies described how global climatic drivers affect wildfires in specific regions, due to the limited hotspots of global wildfire patterns, it is not easy to compare with some climate-wildfire relationships, such as ENSO-wildfire dynamics in Insular Southeast Asia^11^, AO-wildfire in Central Siberia^12^ and AMO-ENSO-PDO-wildfire in Colorado^13^. On the other hand, the advantage of teleconnection between global wildfire patterns and global climatic drivers lies in direct finding the widespread climatic influence from a global perspective, that is, the discovery of global wildfire homogeneity under the influence of the same climatic drivers. Therefore, how to further balance regional phenomena and global relevance should be addressed in future studies.

## Materials and methodology

### Global burned area data

The existing burned area datasets have advantages and limitations. The four candidate global burned area datasets are shown in Table 2. Chuvieco et al.^50^ developed the Fire CCI v5.1 burned area dataset during 2001–2019 based on a hybrid approach that combines a MODIS highest resolution (250 m) near-infrared band and active wildfire information from thermal channels. In addition, Fire CCI v5.1 was considered to perform better than NASA MCD64A1 v006, especially in terms of small wildfire detection capacity^2^. Compared to the GFED 4.1 s and GABAM datasets, Fire CCI v5.1 takes into account the characteristics of long-term and up-to-date data. Therefore, Fire CCI v5.1 was selected as the most suitable dataset.

**Table 2:** Information for the candidate global burned area datasets.

Global burned area datasets	Resolution	Available times
GFED 4.1 s	0.25°	1997/1–2016/12
GABAM	30 m	2000, 2005, 2010, 2015, 2018
Fire CCI v5.1	250 m	2001/11–2019/12
NASA MCD64A1 v006	500 m	2000/11–2020/6

To avoid local variations, we performed the data aggregation and changed the spatial resolution of the original data. Therefore, to obtain better homogeneous burned area results, the Fire CCI v5.1 dataset was processed to a 1° × 1° resolution based on the monthly scale.

### Burned area anomalies analysis

This paper focused on the homogeneity of global wildfire burned areas. If the original burned area time series was directly utilized, the homogeneous region of the burned area would be immoderately dominated by regions with large, burned areas, such as the USA or Canada, making it difficult to explain the climatic causes of homogeneous regions. First, log-transformation was performed for burned area time series. Log transformation is commonly used in the statistical analysis of wildfires (e.g., burned areas)^51–53^ and could increase attention to wildfire-sensitive ecosystems that are rarely affected by wildfires and have a lower restoration capability of vegetation than other wildfire-dependent ecosystems. The monthly log-transformed burned area anomalies (logBAA) were subsequently calculated, as shown in Formula 1.

1 $$\log BAA_{i,n} = \log BA_{i,n} - \frac{{\sum\nolimits_{n = 1}^{m} {\log BA_{i,n} } }}{m}$$

where log*BA*_*i,n*_ is the monthly log-transformed wildfire burned area in month *i* of year *n* in a given mesh; *m* is the number of years in the study period; and log*BAA*_*i,n*_ is the monthly log-transformed wildfire burned area anomaly in month *i* of year *n* in a given mesh.

## Global climatic drivers

In this paper, sixteen global climatic drivers that may have impacts on wildfires were selected, as shown in Table 3. To explore the diversity of climate causes, both oceanic indices such as the Oceanic Niño Index and continental teleconnection patterns such as the East Atlantic/Western Russia Pattern were considered. This paper selected as many global climatic drivers as possible to assess the complex underlying causes of wildfires. These global climatic drivers were sourced from the National Oceanic and Atmospheric Administration (NOAA): https://www.noaa.gov/ (last access: 11 May 2021).

**Table 3:** Description and key references of sixteen global climatic drivers.

Global climatic drivers	Abbreviation	Primitive elements	Key references
Polar/Eurasia Pattern	POL	Primitive element: geopotential height field	Barnston et al.^54^
Dipole Mode	DMI	Primitive element: sea surface temperature	Saji et al.^55^
Arctic Oscillation	AO	Primitive element: sea level pressure	Thompson et al.^56^
Antarctic Oscillation	AAO	Primitive element: geopotential height field	Gong et al.^57,58^
Western Pacific Pattern	WP	Primitive element: geopotential height field	Wallace et al.^59^ Barnston et al.^54^
East Atlantic/Western Russia Pattern	EA/WR	Primitive element: geopotential height field	Barnston et al. ^54^
Pacific/North American Pattern	PNA	Primitive element: geopotential height field	Blackmon et al.^60^
Pacific Decadal Oscillation	PDO	Primitive element: sea surface temperature	Newman et al^61^
East Pacific/North Pacific Oscillation	EP/NP	Primitive element: geopotential height field	Bell et al.^62^
Multivariate ENSO Index	MEI	Primitive elements: sea level pressure, sea surface temperature, surface zonal winds, surface meridional winds, and outgoing longwave radiation	Wolter et al.^63–65^
Oceanic Niño Index	ONI	Primitive element: sea surface temperature	Huang et al.^66^
Atlantic multidecadal Oscillation	AMO	Primitive element: sea surface temperature	Enfield et al.^67^
North Atlantic Oscillation	NAO	Primitive element: sea level pressure	Wallace et al.^59^ Barnston et al.^54^ Hurrell^68^
East Atlantic Pattern	EA	Primitive element: geopotential height	Wallace et al.^59^ Barnston et al.^54^
Tropical Northern Atlantic Pattern	TNA	Primitive element: sea surface temperature	Enfield et al.^69^
Tropical Southern Atlantic Pattern	TSA	Primitive element: sea surface temperature	Enfield et al.^69^

### Distinct empirical orthogonal function

The empirical orthogonal function (EOF), which deals with temporal and spatial functions, is used to extract the spatiotemporal modes based on the data variance representations. EOF was introduced into meteorology and climate research by Lorenz^70^ in the 1950s and has already been widely applied in other fields, such as geoscience and hydrology. The EOF analysis method can decompose the time-varying variable fields into the space function part (EOFs) that does not change with time and the time function part (principal components, PCs) that depends only on time. DEOF analysis was subsequently introduced to overcome problems in EOF analysis^71^. In the DEOF, a continuous spectrum of spatial patterns resulting from a stochastic process can be represented by DEOF modes, where some spatial structures will be more dominant than others. Based on the isotropic diffusion null hypothesis, DEOFs can be found by rotating the leading EOF modes, corresponding to the distinguished principal components (DPCs)^72^. These DPCs take up a large part of the total variance in all the variables in the original field, which is equivalent to the main information of the original field concentrated on a few main components. The details about DEOF can be found in Dommenget^71^.

### Wavelet analysis

The continuous wavelet transform (CWT)^73^ is widely used for analyzing the frequency domain of hydrometeorological time series^74,75^. The spectral and temporal features of the time series can be projected onto a time–frequency plane by CWT, where the dominant cycle period and its duration can be identified^76^. The square modulus of the CWT defines the wavelet power spectrum (WPS)^77^, which represents the signal energy at a specific scale (period) and time^78^. In this paper, the time–frequency domain of DPCs was analyzed by CWT. The specific calculation process for the CWT can be found in Torrence et al.^73^. Notably, CWT brings about a cone of influence (COI) that delimits a region of the WPS beyond which the edge effects become significant, which means that outcomes outside COI should be suspected^73^.

Additionally, the cross wavelet transform (XWT) ^73^ and wavelet coherence (WCO)^79^ can examine the relationship between the DPCs and the global climatic driving factor. WCO reveals local similarities between two time series and may be found to be a local correlation coefficient in the time–frequency plane; that is, their possible teleconnection can be identified by WCO^78^. Similar to CWT, the parts outside of the COI should also be interpreted with caution. The specific XWT and WCO analysis methods can also be found in Torrence et al.^73,79^. The wavelet coherence coefficient was defined by R^2^^73,79^, which takes a value between 0 and 1, where 0 indicates no correlation between the two time series and 1 indicates that the two time series are perfectly correlated with each other^73,79^.

For evaluating multivariate coherence, multiple wavelet coherence (MWC)^80^ was utilized. MWC was developed to untangle multivariate coherences. It can determine the proportion of the variance associated with a response variable explained by predictor variables and identify spatiotemporal scale multivariate coherences^19^. Additionally, a significance level of *p* < 0.05 was used to evaluate the statistical significance of the results. To measure the extent of climatic influence on wildfire burned area anomalies, the percent area of significant coherence (PASC) relative to the wavelet scale-location domain was adopted^80^. The larger the PASC is, the greater the average coherence is, indicating that more of the wildfire variation can be explained. The global wavelet coherence coefficient^81^ was defined to evaluate the coherence between two time series at different scales while neglecting the influence of time. PASC is used to identify the most significant coherent variable, and the global wavelet coherence coefficient is used to quantitatively judge the level of coherence. Considering all scales of wildfire variation could help us understand the factors underlying the variations in wildfires.

## Data Availability

All datasets utilized to perform this study are freely available on the internet. For further information, please contact the corresponding author. The specific data sources are as follows: Global burned area data: https://geogra.uah.es/fire_cci/firecci51.php. Global climatic driver data: https://psl.noaa.gov/data/climateindices/list/. Shape for countries of the world: http://tapiquen-sig.jimdofree.com. Land cover data: https://land.copernicus.eu/global/products/lc.
